# The protective role of IL-17C in oral squamous cell carcinoma

**DOI:** 10.1016/j.tranon.2025.102541

**Published:** 2025-09-19

**Authors:** Zivile Giedraityte, Joosua Suominen, Wafa Wahbi, Ahana Kapuge Dona Varuni Yashodha Ratnayake, Tuulia Onali, Martina Giacomini, Sami Kilpinen, Katja Korelin, Maija Risteli, Tuula Salo, Ahmed Al-Samadi

**Affiliations:** a Department of Oral and Maxillofacial Diseases, Clinicum, Faculty of Medicine, University of Helsinki, Helsinki, Finland; b Institute of Dentistry, School of Medicine, Faculty of Health Sciences, University of Eastern Finland, Kuopio, Finland; c Translational Immunology Research Programme (TRIMM), University of Helsinki, Helsinki, Finland; d Molecular and Integrative Biosciences Research Programme, Faculty of Biological and Environmental Sciences, University of Helsinki, Helsinki, Finland; e Research Unit of Population Health, Faculty of Medicine, University of Oulu, Oulu, Finland; f Medical Research Centre, Oulu University Hospital, University of Oulu, Oulu, Finland; g Department of Pathology, HUSLAB, Helsinki University Central Hospital, University of Helsinki, Helsinki, Finland; h iCAN Digital Precision Cancer Medicine Flagship, University of Helsinki, Helsinki, Finland

**Keywords:** Oral cancer, IL-17C, Cytokine, Zebrafish, Proliferation

## Abstract

**Background:**

Oral squamous cell carcinoma (OSCC) is a common cancer characterised by high rates of metastasis and mortality. Interleukin‑17C (IL-17C) is expressed by several cell types, including epithelial cells, and signals through the heterodimeric IL-17RE/IL-17RA receptor complex at various mucosal sites. The role of IL-17C in OSCC development and progression remains unexamined; therefore, this study aimed to elucidate its potential involvement.

**Methods:**

Among 11 OSCC cell lines, OU-OTSCC-7B and UH-SCC-17B exhibited a high transcript expression of IL-17C receptors (IL-17RE and IL-17RA) and were selected for further assays. Proliferation, apoptosis, migration, and invasion assays were conducted in the presence and absence of IL-17C. Using a zebrafish xenograft model, we assessed the *in vivo* effects of IL-17C. Additionally, we performed RNA sequencing after stimulating UH-SCC-17B cells with IL-17C.

**Results:**

IL-17C significantly inhibited the proliferation and migration of UH-SCC-17B cells, but had no effect on OU-OTSCC-7B cells. Moreover, IL-17C reduced the tumour size of UH-SCC-17B cells by approximately 10% in the zebrafish model. RNA sequencing revealed the downregulation of five genes, three of which were mitochondrial genes—MT-CYB, MT-ND1, and MTND2P28—implicated in cancer progression. Gene set enrichment analysis revealed the enrichment of the MYC target and oxidative phosphorylation gene sets following IL-17C treatment.

**Conclusions:**

IL-17C appears to play a protective role in some OSCC cells. While this study indicates the potential of using IL-17C as a therapeutic candidate for OSCC patients, its relatively mild and selective effect suggests it may be more effective as part of a combination therapy.

## Highlights

• The role of IL-17C in OSCC development and progression remains unexamined.
• IL-17C suppressed OSCC tumour growth both *in vitro* and *in vivo*.
• IL-17C downregulated the expression of MTND2p28, TUBA1A, MT-CYB, MT-ND1, and H19.
• IL-17C enriched MYC_TARGET_V1 and OXIDITAVE_PHOSPHORYLATION pathways.
• IL-17C could serve as a promising targeted therapy for a subset of OSCC patients.

## Introduction

Head and neck squamous cell carcinoma (HNSCC) is a group of cancers that develop in the oral and nasal cavity, pharynx, and larynx. Despite advances in cancer research, HNSCC remains one of the most aggressive cancers, with a poor prognosis and high mortality rate, causing more than 458 000 deaths globally in 2022 [1,2]. Oral squamous cell carcinoma (OSCC) constitutes the largest subgroup of HNSCC, with oral tongue squamous cell carcinoma (OTSCC) its most prevalent form [2]. Although targeted therapies and immunotherapies, such as EGFR and PD-1 antibodies, have been approved for treating recurrent and metastatic HNSCC, the five-year survival rate has not shown significant improvement and remains around 50 % [3,4].

Cytokines are signalling molecules that mediate molecular communication, enabling coordinated yet self-limited immune responses against target antigens [5]. In addition to their critical role in regulating cellular interactions between tumours and the immune system [6] cytokines also play a crucial role in tumour development and progression, exerting both pro-tumorigenic and antitumorigenic effects [7,8]. Given their central role in cancer biology, cytokines such as interleukin (IL)-12, IL-15, and IL-21 are currently under clinical evaluation, highlighting their therapeutic relevance [5]. This has been particularly evident in melanoma, where IL-2 and IL-15 have been shown to upregulate activating receptors on lymphocyte subsets and enhance the antitumor activity of immune cells within regional lymph nodes [9,10]. Cytokine-based immunotherapy has emerged as a promising treatment strategy, demonstrating efficacy in several cancer types, particularly in modulating tumour activity in skin cancer. Consequently, cytokine-based immunotherapy may also hold therapeutic potential for OSCC [11].

Among the cytokines involved in tumour progression and inflammatory responses is the IL-17 family, which comprises six members: IL-17A through IL-17F [12]. IL-17A, the first member identified, is often referred to simply as IL-17 [13,14]. While IL-17A has been extensively studied, the role of other IL-17 family members remains largely unexplored. IL-17A, a pro-inflammatory homodimeric glycoprotein composed of 155 amino acids, plays a key role in host defence, cancer progression, and inflammatory conditions [12,15]. In OTSCC, IL-17A appears to have pro-tumorigenic effects, although, in some contexts, may also exhibit antitumorigenic properties [16,17]. Another closely related cytokine, IL-17F, reportedly exerts protective effects against various cancer types, including HNSCC and pancreatic cancer [18]. Previously, we demonstrated that, in OTSCC, IL-17F has antitumorigenic effects by inhibiting cancer cell proliferation, migration, and angiogenesis [[19], [20], [21]].

Among the lesser-studied members of the IL-17 family, IL-17C remains one of the least explored, with its role in cancer progression is poorly understood. IL-17C signals through a heterodimeric receptor complex comprising IL-17RA and IL-17RE. IL-17RA is a shared receptor also utilised by IL-17A, IL-17F, and IL-17E, whereas IL-17RE is a unique subunit that provides high-affinity recognition and selectivity for IL-17C [[22], [23], [24]]. A high expression of IL-17C and its receptors has been reported in human oral epithelial cells in recurrent aphthous ulcers [25]. Notably, unlike most IL-17 family cytokines produced by immune cells, IL-17C is also secreted by epithelial cells [25]. This distinction underscores the need to investigate its role in OSCC and its potential involvement in tumour development.

Recent studies suggest that IL-17C may have both pro- and antitumorigenic functions, depending upon cancer type. For instance, in non-small cell lung cancer, IL-17C promotes tumour growth by mediating neutrophil recruitment [26]. Conversely, in liver cancer, serum IL-17C acts as a protective factor [27]. However, its function in OSCC remains unclear, highlighting the importance of elucidating its role in cancer progression. This study aims to evaluate the effects of IL-17C on OSCC progression through *in vitro* and *in vivo* assays.

## Methods

### Transcriptome analysis of IL-17RE and IL-17RA from GSE103322

Data from GSE103322 were downloaded from the URL pointed out in the GEO record [28]. Cross-comparison with the publication of GSE103322 revealed data fell on the log2(TPM/10+1) scale.

The cell annotation used in the UMAP plot was simplified such that the cell type given by the original authors was the primary annotation. However, since that was given only for non-cancer cells, we also took binary non-cancer/cancer information provided by the original authors and assigned the cell type ‘cancer’ to the remaining cells. Cells from metastases (lymph node = 1) were dropped from the calculation leading to the UMAP, but not from boxplots.

To calculate the UMAP, data were returned to the log1p scale allowing for the entire dynamic range of the transcriptome contributing to the UMAP topology. The dataset was first transformed through PCA, and 1:10 components of the PCA were then subjected to the UMAP calculation. Nearest neighbour or clustering was not performed since we intended to project existing cell level annotation to the plot and subsequently visualise the expression of the genes of interest on top of it.

### OSCC cell lines

Eleven locally established OSCC cell lines were used in this study (Table 1). Seven cell lines (UH-SCCs) were established in the Department of Oral and Maxillofacial Diseases at the University of Helsinki (Helsinki, Finland) and four cell lines (OU-SCC and OU-OTSCCs) at Oulu University Hospital (Oulu, Finland) using the protocol described by Tuomainen et al. [29] The cells were cultured using Dulbecco’s Modified Eagle Medium/Nutrient Mixture F-12 (DMEM-F12; Gibco, Paisley, UK) supplemented with 10 % foetal bovine serum, 100-U/ml penicillin, and 100-μg/ml streptomycin (Merck, Darmstadt, Germany) in T-75 flasks. The cancer samples used to establish the OSCC cell lines were collected following institutional research ethics board approval (Regional Ethics Committee of Northern Ostrobothnia Hospital District, statement number 31/2016). Patient participation was voluntary and required informed consent.

**Table 1:** Clinical and pathological characteristics of the established OSCC cell lines from primary and metastatic sites.

Cell line	Gender^A^	Age^B^	TNM	Specimen site	Primary tumour site	Type^C^	Grade
**UH-SCC-9**	M	58	pT2pN3b	Mobile tongue	Mobile tongue	Pri	G2
**UH-SCC-13**	M	65	T2N0	Tongue#	Tongue#	Pri	G3
**UH-SCC-17A**	M	50	T3N2bM0	Mobile tongue	Mobile tongue	Pri	G2
**UH-SCC-17B**	M	50	T3N2bM0	Neck	Mobile tongue	Met	G2
**UH-SCC-20A**	M	75	T4aN3bM0	Lower gum	Lower gum	Pri	G2
**UH-SCC-20B**	M	75	T4aN3bM0	Neck	Lower gum	Met	G2
**UH-SCC-23B**	F	69	T3N1M0	Neck	Cheek mucosa	Met	G3
**OU-OTSCC7B**	M	72	T3N2cM0	Neck	Mobile tongue	Met	G2
**OU-SCC-9B**	M	54	T4N3M1a	Neck	Mobile tongue	Met	G2
**OU-OTSCC-18A**	M	55	T3N3M0	Mobile tongue	Mobile tongue	Pri	G2
**OU-OTSCC-18B**	M	55	T3N3M0	Neck	Mobile tongue	Met	G2

A M, male; F, female.

B Age in years.

C Pri, primary tumour; Met, lymph node metastasis.

# Unspecified parts of the tongue.

### Quantitative real-time PCR

To investigate the expression of the IL-17C receptors—IL-17RE and IL-17RA—in OSCC cells, total RNA was purified from each cell line using the RNeasy Mini Kit (Qiagen, Düsseldorf, Germany). We used 200-ng RNA for cDNA synthesis employing the iScript cDNA Synthesis Kit (Bio-Rad, Hercules, *CA*, USA). For PCR, 10 μl of Fast SYBR™ Green Master Mix (Thermo Scientific, MA, USA), 7 μl of water, and 1 μl of a 250 nM primer solution were added to 2 μl of cDNA. Glyceraldehyde 3-phosphate dehydrogenase (GAPDH) was used as the housekeeping gene. The primer sequences are listed in Supplemental Table 1.

**Fig. 1:**
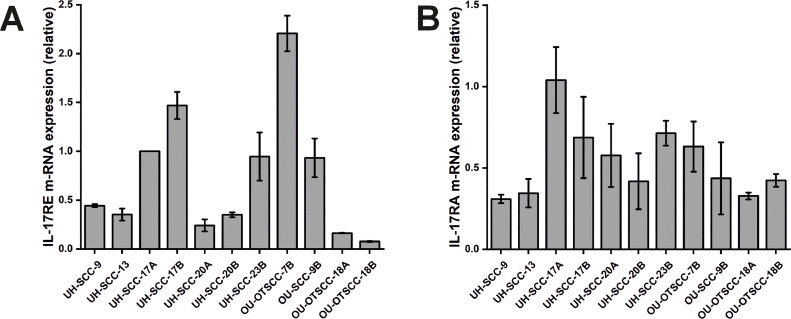
Expression of IL-17C Receptors in 11 OSCC cell lines. (A) OU-OTSCC-7B and UH-SCC-17B showed the highest expression of IL-17RE, (B) and the fourth and third highest expression of IL-17RA, respectively. Data are presented as means ± standard deviations.

OU-OTSCC-7B and UH-SCC-17B exhibited the highest IL-17RE expression and the fourth and third highest IL-17RA expression levels, respectively (Fig. 1). Therefore, these cell lines were selected for subsequent assays.

### Luminescent cell viability assay

For the assay, we used 96-well plates with black walls and clear bottoms (Essen Bioscience, Ann Arbor, MI, USA). A total of 1000 OU-OTSCC-7B and UH-SCC-17B cells were seeded per well and allowed to adhere overnight. IL-17C (R&D, Minneapolis, MN, USA) was added at final concentrations of 10, 50, and 100 ng/ml, and the cells were incubated for 72 h.

After incubation, the plates were left at room temperature for 15 min. Then, 100 μL of CellTiter-Glo (Promega, Madison, WI, USA) was added to each well. The plates were placed on a plate shaker (Heidolph, Schwabach, Germany) for 5 min at 450 rpm, followed by 5 min in a plate spinner (Thermo Scientific) at 1000 rpm. Finally, luminescence was measured using the BMG PHERAstar FS plate reader (BMG Labtech, Offenburg, Germany) to assess cell viability.

### Apoptosis assay

OU-OTSCC-7B and UH-SCC-17B cells were stained with CellTrace Far Red (Invitrogen, Carlsbad, *CA*, USA) and seeded into 96-well plates (Corning, NY, USA) at a density of 2000 cells per well, then allowed to adhere overnight. The following day, the culture medium was replaced with medium containing IL-17C (R&D Systems) at concentrations of 10, 50, or 100 ng/ml, together with the IncuCyte Caspase-3/7 Apoptosis Assay Reagent (2.5 μM; Sartorius, Göttingen, Germany) for apoptosis detection. Cells were then placed in the IncuCyte S3 Live-Cell Analysis System (Sartorius) and imaged every three hours for two days. The number of apoptotic cells (green object count) and the ratio of apoptotic cells (green and red objects divided by red objects) were quantified using IncuCyte software.

### Scratch wound cell migration assay

OU-OTSCC-7B and UH-SCC-17B cells were seeded at a density of 25 000 cells per well in an IncuCyte 96-well Image Lock Microplate (Sartorius) and incubated overnight. On the next day, a wound maker (Sartorius) was used to create uniform scratch wounds. Wounds were examined under a light microscope, and the culture medium was replaced with fresh medium supplemented with IL-17C (R&D) at 10, 50, and 100 ng/ml.

The plates were placed in an IncuCyte incubator (Sartorius), and wound confluence was monitored using the IncuCyte Live-Cell Imaging System (Sartorius). Images were captured hourly for 36 hours.

### Spheroid invasion assay

OU-OTSCC-7B and UH-SCC-17B cells were seeded at a density of 1000 cells per well in 100 μL of medium in ultra-low attachment 96-well round-bottom plates (Corning). The plates were incubated for four days to allow spheroid formation.

Successful spheroids were then embedded in 100 μL of Myogel–fibrin with or without IL-17C (R&D) at 10, 50, and 100 ng/ml. The Myogel–fibrin mixture was prepared with the following components: 0.5-mg/ml Myogel (lab-made [30]), 0.5-mg/ml fibrinogen (Merck), 0.3-U/ml thrombin (Merck), and 33.3-µg/ml aprotinin (Merck). The gels were allowed to solidify for 30 min before adding 100 μL of DMEM-F12 to each well.

The plates were incubated for four days, and images were taken daily using a Nikon Digital Sight DS-U3 microscope (Nikon, Tokyo, Japan) at 4x magnification. Cancer cell invasion was analysed using the ImageJ software (Wayne Rasband, National Institute of Mental Health, Bethesda, MD, USA), following the protocol described by Naakka et al. [31].

### Zebrafish xenograft assay

Two-day-old wild-type zebrafish (AB strain) were dechorionated, anesthetised with 0.04 % Tricaine, and microinjected with 4 nl of CellTrace Far Red (Invitrogen) stained UH-SCC-17B cell suspension (1000 cells) in the perivitelline space. The larvae were then transferred to a fresh embryonic medium with or without 100 ng/ml IL-17C (R&D), in a 24-well plate and incubated at 34 °C for 72 h. After incubation, the zebrafish were fixed with 4 % PFA, mounted on glass slides, and imaged under a Nikon Ti-E fluorescence microscope. Tumour size was measured using the ImageJ software. We used 30 zebrafish for each group in each experiment.

### RNA sequencing

To identify the molecular mechanisms underlying the effect of IL-17C on UH-SCC-17B cells, we conducted RNA sequencing (RNA-seq). UH-SCC-17B cells were stimulated with 100 ng/ml IL-17C in triplicates *versus* controls for 24 h and then total RNA was extracted using a RNeasy Mini Kit (Qiagen) according to the manufacturer instructions. The quality of total RNA was assessed with a TapeStation (Agilent Technologies, Santa Clara, *CA*, USA), and only samples of high quality (RNA integrity value >8) were included in the analyses. RNA-seq was completed at Novogene (Novogene GmbH, Munich, Germany). A non-strand-specific library was prepared as follows: 1) mRNA purified using poly-T oligo-attached magnetic beads; 2) fragmentation consisting of cDNA synthesis, end repair, adapter ligation, size selection, amplification, and purification; and 3) quality check completed with Qubit, real-time PCR, and Bioanalyzer. For clustering and sequencing, different libraries were pooled based on the effective concentration and targeted data amount, then subjected to Illumina sequencing. Clean readings were obtained by removing reads containing adapter, reads containing ploy-N, and low-quality reads from raw data. At the same time, Q20, Q30, and GC content with the clean data were calculated. The index of the reference genome was built using Hisat2 v2.0.5 and paired-end clean reads were aligned to the reference genome using Hisat2 v2.0.5. Quantification of the gene expression level feature relied on Counts v1.5.0-p3 to count the read numbers mapped to each gene and then the FPKM of each gene was calculated based on the length of the gene and read counts mapped to this gene. The differential expression analysis for two conditions (IL-17C stimulated samples *vs.* controls) was performed using the DESeq2 R package (1.20.0). The resulting P-value was adjusted using Benjamini and Hochberg’s methods to control the error discovery rate. The corrected P-value ≤ 0.05 and |log2(foldchange)| ≥ 1 was set as the threshold of significant differential expression. Prior to differential gene expression analysis, for each sequencing library, read counts were adjusted using the edgeR R package (3.22.5) by scaling normalisation factors to eliminate differences in sequencing depth between samples, followed by differential expression analysis.

Gene set enrichment analysis (GSEA) was performed for triplicate samples of IL-17C stimulated *versus* controls as described in Subramanian et al. [32]. Enrichment statistics were computed for gene sets from MSigDB’s Hallmarks collection (http://www.broad.mit.edu/gsea/msigdb/) version2024.1.Hs (h.all.v2024.1.hs.symbols.gmt) in the gene_set permutation mode. Gene identifiers were collapsed to symbols using the MSigDB-version matched annotation file: Human_Ensembl_Gene_ID_MSigDB.v2024.1.Hs, employing the collapse mode. The random seed value derived from the system timestamp was used to initialise the permutation matrix.

### Statistical analysis

Statistical analyses were performed using SPSS (version 29.0; IBM Corp., Armonk, NY, USA) and figures were created using OriginLab (OriginLab Corporation, Northampton, MA, USA). All experiments were conducted independently three to four times, each in duplicate or triplicate. Data are presented as means ± standard deviations.

To assess statistical significance in proliferation, apoptosis, migration, and invasion assays, we performed one-way analysis of variance (ANOVA) followed by the Bonferroni post-hoc test. For migration and apoptosis assays, the area under the curve (AUC) was calculated, and the resulting values were used for statistical analysis. In the zebrafish assay, the average tumour area from all fish in each experiment was calculated and analysed using a paired-sample *t*-test. Statistical significance was set at *P* ≤ 0.05.

## Results

### IL-17C and its receptors’ (IL-17RE and IL-17RA) expression in HNSCC

**Fig. 2:**
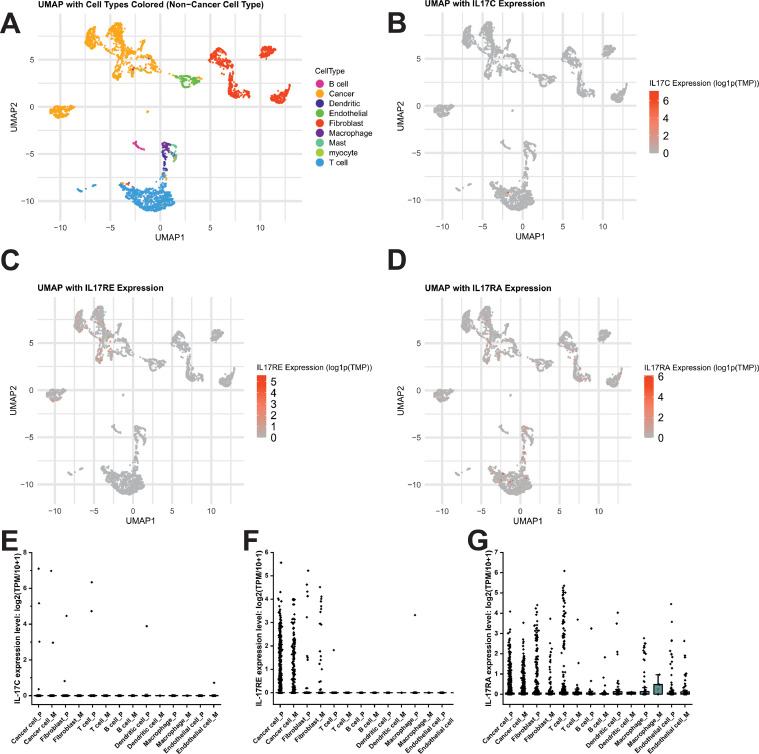
Single-cell RNA-seq Analysis of IL-17C and Its Receptor Components in HNSCC Tumours. (A) UMAP plot of primary HNSCC samples with the cell types colour-coded. (B–D) UMAPs showing the expression levels of IL-17C (B), IL-17RE (C), and IL-17RA (D) across the cell populations. (E–G) Expression levels of IL-17C (E), IL-17RE (F), and IL-17RA (G) in various cell types, comparing primary tumours (P) and metastatic lymph node samples (M).

To investigate the cellular sources of IL-17C signalling components in HNSCC tumours, we analysed a single-cell RNA-seq dataset from the study by Puram et al. [28]. The IL-17C expression was nearly absent across all major cell types in both primary and metastatic tumour samples (Fig. 2A, 2B and 2E). By contrast, IL-17RE was predominantly expressed in malignant cells, followed by the fibroblasts, with comparable expression patterns in primary and metastatic samples (Fig. 2C and 2F). IL-17RA showed broad expression across multiple cell types, including malignant cells, fibroblasts, and immune populations, although at varying expression levels (Fig. 2D and 2G).

### The effect of IL-17C on OSCC cell proliferation, apoptosis, migration, and invasion

**Fig. 3:**
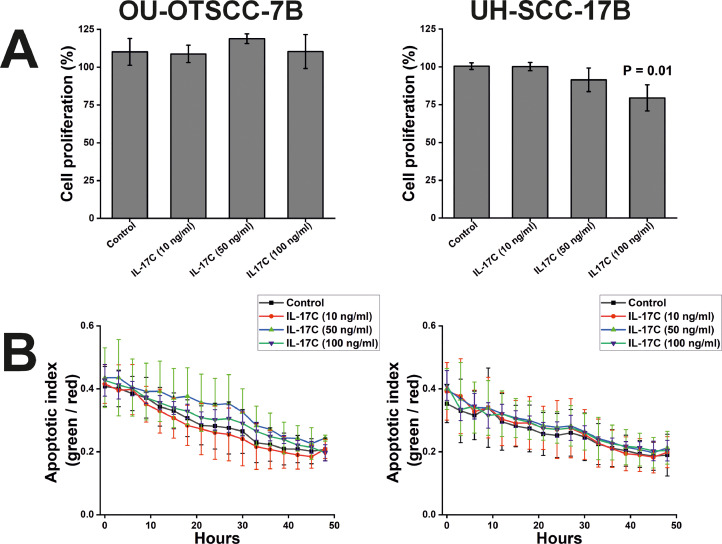
Effects of IL-17C on OSCC Cell Proliferation, and Apoptosis. (A, B) Treatment with IL-17C had no effect on the proliferation and apoptosis of OU-OTSCC-7B cells (left-hand side). (A, B) By contrast, IL-17C significantly suppressed the proliferation but not apoptosis of UH-SCC-17B cells (right-hand side). Each experiment was conducted at least three times independently. Data are presented as means ± standard deviations.

To determine whether IL-17C influences OSCC cell proliferation, apoptosis, migration, and invasion, we conducted a cell viability assay, an IncuCyte apoptosis assay, a wound migration assay, and a spheroid invasion assay, respectively. IL-17C inhibited the proliferation of UH-SCC-17B cells in a dose-dependent manner, reaching statistical significance at a concentration of 100 ng/ml (Fig. 3A). By contrast, we observed no inhibition of proliferation in OU-OTSCC-7B cells (Fig. 3A). On the other hand, IL-17C had no effect on apoptosis in either cell line (Fig. 3B).

**Fig. 4:**
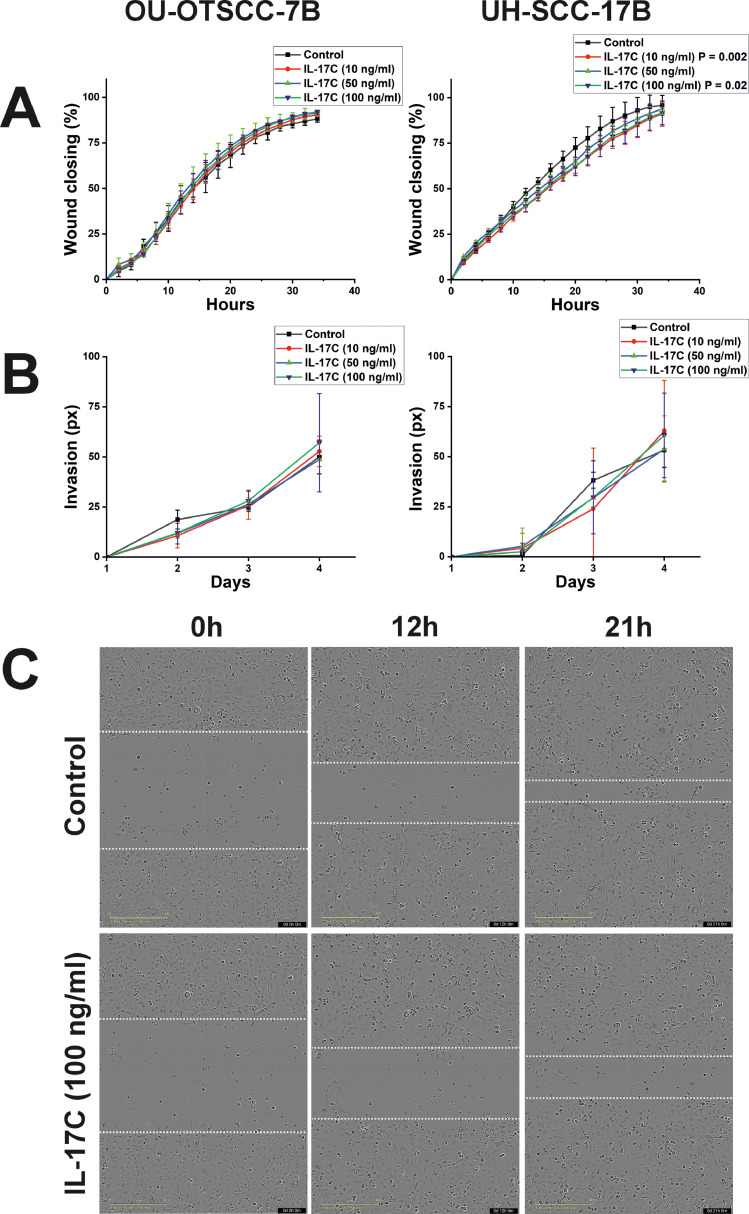
Effects of IL-17C on OSCC Migration, and Invasion. (A, B) IL-17C treatment had no effect on the migration or invasion of OU-OTSCC-7B cells (left-hand side). In contrast, IL-17C significantly suppressed the migration, but not the invasion, of UH-SCC-17B cells (right-hand side). (C) Representative images from an IncuCyte wound migration assay showing the effect of IL-17C (100 ng/ml) on the wound closure of UH-SCC-17B Each experiment was conducted at least three times independently. Data are presented as means ± standard deviations.

Consistent with the proliferation result, IL-17C reduced the migration of UH-SCC-17B cells at all doses tested, with significant inhibition observed at 10 and 100 ng/ml. However, no effect was detected on the OU-OTSCC-7B cells (Fig. 4A and 4C).

In comparison to the proliferation and migration assays, IL-17C did not influence the OSCC cell invasion regardless of the cell line and cytokine concentration (Fig. 4B).

### The effect of IL-17C on OSCC tumour size in the zebrafish larvae xenograft model

**Fig. 5:**
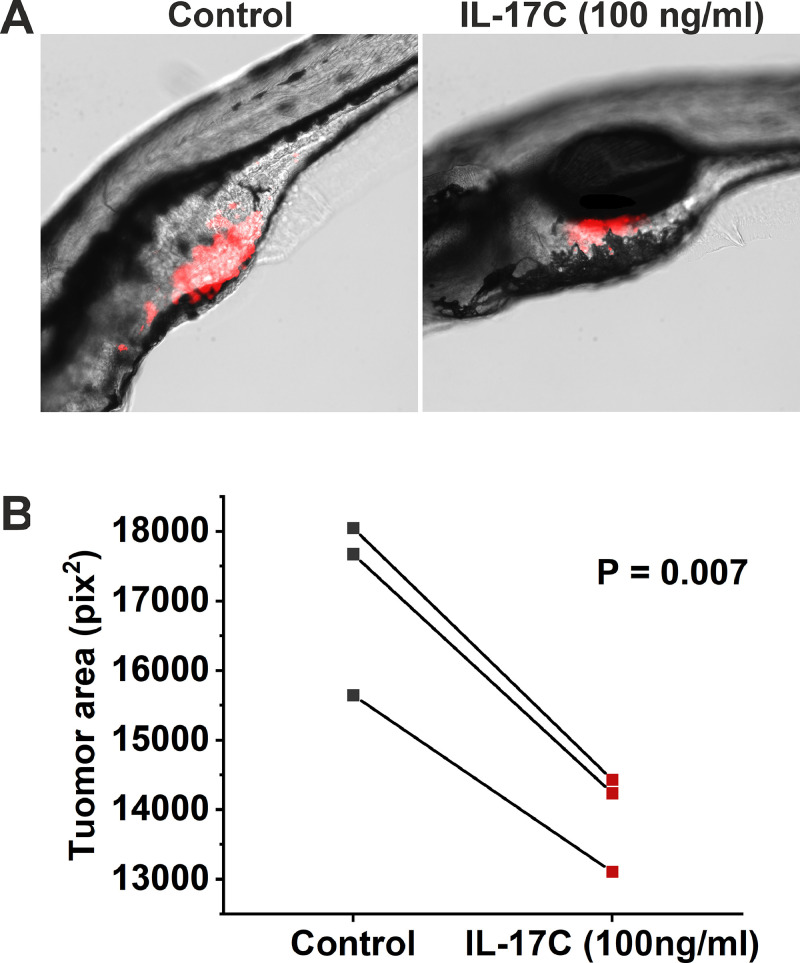
The Effect of IL-17C on Tumour Size in the Zebrafish Larvae Xenograft Model. UH-SCC-17B cells were labelled with CellTrace Far Red and injected into zebrafish larvae. The zebrafish larvae were then incubated for three days with or without 100 ng/ml of IL-17C. (A) Representative images showing the tumour (red) inside the zebrafish after three days of treatment. (B) IL-17C treatment (100 ng/ml) significantly reduced the tumour size compared with the control group. Data are presented as the mean tumour area of all zebrafish in each experiment.

To verify the effect of IL-17C on UH-SCC-17B growth *in vivo*, we conducted a zebrafish larvae xenograft assay. Consistent with the *in vitro* results, treatment with 100 ng/ml of IL-17C significantly reduced the tumour size of UH-SCC-17B by approximately 10 % (Fig. 5). However, no effect on cancer cell metastasis was detected (data not shown).

### Transcriptome analysis and GSEA analysis

**Fig. 6:**
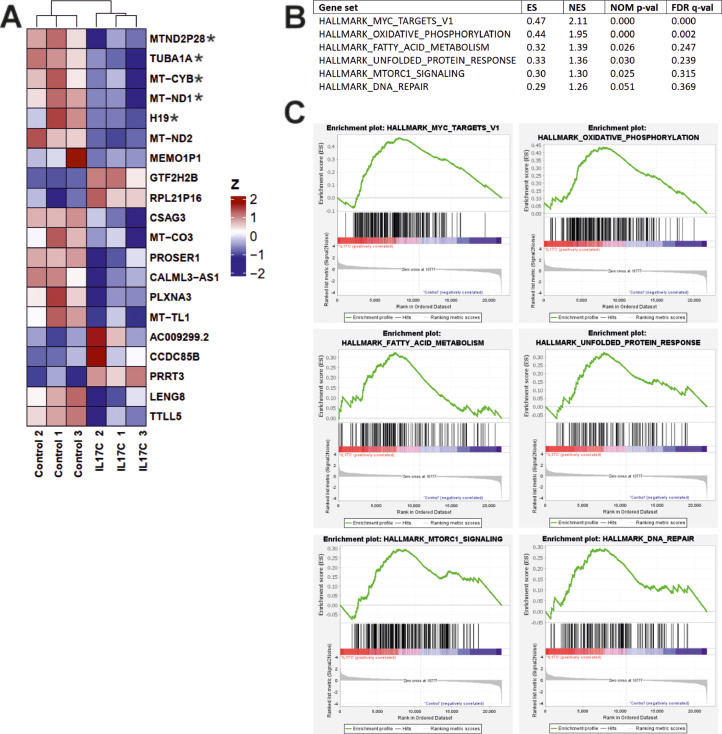
**Transcriptome Analysis and GSEA Results.** (A) Heatmap showing the top 20 differentially expressed genes in OU-SCC-17B cells after stimulation with 100 ng/ml IL-17C. (B) The top five enriched Hallmark pathways following IL-17C stimulation. (C) Enrichment plot depicting the enrichment score *versus* ranked gene abundance. *Genes that reached statistical significance. ES: Enrichment Score. NES: Normalized Enrichment Score.

To investigate the underlying mechanisms of tumour suppression following IL-17C stimulation, we performed transcriptome analysis to identify differentially expressed genes. While several genes were affected by IL-17C stimulation, only five reached statistical significance, and all were downregulated (Fig. 6A). The two most upregulated genes in response to IL-17C were pseudogenes: general transcription factor IIH subunit 2B (GTF2H2B) and ribosomal protein L21 pseudogene 16 (RPL21P16).

To further understand the signalling pathways involved in the IL-17C-mediated inhibition of cancer cell proliferation, we performed GSEA. Among the top six enriched gene sets, only two reached statistical significance at FDR q-val < 0.05: MYC_TARGET_V1 and OXIDITAVE_PHOSPHORYLATION (Fig. 6B and C).

## Discussion

IL-17C is a 15–20-kDa glycosylated cytokine that plays a crucial role in mucosal immunity, with potential pro-tumour or antitumour effects [14,33]. It is expressed by several cells including epithelial cells and functions through the heterodimeric IL-17RE/A receptor complex [33]. While other IL-17 family members, such as IL-17A and IL-17F, have been well-studied in OSCC, the role of IL-17C remains poorly understood. In this study, we demonstrated for the first time that IL-17C exerts antiproliferative and antimigratory effects on some metastatic OSCC cells, as shown through *in vitro* and *in vivo* assays.

Since IL-17C binds to the heterodimeric IL-17RE/A receptor complex [33], we first examined the expression of IL-17RA and IL-17RE in 11 OSCC cell lines. Whilst all cell lines expressed both receptors, we selected OU-OTSCC-7B and UH-SCC-17B for further analysis, because they showed the highest receptor expression levels. Notably, both cell lines are from neck lymph node metastasis, and their primary sites were the mobile tongue of male patients. Additionally, we analysed the expression of IL-17C and its receptors in HNSCC using single-cell RNA sequencing. Our data revealed that IL-17C is minimally expressed in HNSCC; however, both of its receptors are expressed at varying levels in HNSCC cancer cells, suggesting that these cells could serve as suitable targets for IL-17C stimulation.

Interestingly, like IL-17F, IL-17C significantly reduced UH-SCC-17B cell proliferation and migration, although it did not impact apoptosis and invasion [20]. This effect contrasts with previous findings in lung cancer [26,34], where IL-17C had a pro-tumorigenic role. However, IL-17 family members are known to exhibit context-dependent roles in different cancer types [18,35]. In addition, lung cancer studies used IL-17C knockout (KO) mouse models, rather than direct *in vitro* proliferation and migration assays, eliminating confounding factors and providing insight into the direct role of IL-17C [26,34]. Furthermore, IL-17C did not affect the proliferation, apoptosis, migration, or invasion of OU-OTSCC-7B cells. While the exact reason for these differential responses remains unclear, intrinsic heterogeneity among cell lines may explain the variability in IL-17C sensitivity. Importantly, our apoptosis assays indicated that the inhibitory effect of IL-17C on cell proliferation was not due to increased apoptosis, a phenomenon previously reported with other cytokines such as TNF-α [36].

Consistent with our *in vitro* findings, the zebrafish xenograft assay demonstrated that IL-17C significantly reduced tumour size by approximately 10 %, mirroring the effect observed *in vitro*. While this result confirms and validates our *in vitro* data, it also suggests that the effect of IL-17C is relatively mild. Therefore, if considered for future therapeutic applications, IL-17C would likely be more effective as part of a combination therapy rather than a standalone treatment. Further supporting our *in vitro* results, no effect on cancer cell metastasis was detected in the zebrafish model, aligning with the lack of an impact on cancer cell invasion observed *in vitro*.

Because little is known about the downstream signalling pathway of IL-17C, we sought to understand the mechanism by which IL-17C reduces OSCC cell proliferation and migration. To this end, we performed RNA sequencing and gene expression analysis on UH-SCC-17B cells following IL-17C stimulation. Our results revealed five significantly differentially expressed genes, all of which were downregulated. Among these, three were mitochondrial genes: MT-CYB, MT-ND1, and MTND2P28.

Interestingly, mitochondrial DNA (mtDNA) alterations have been shown to influence cancer cell proliferation, progression, metastasis, and interactions with both the immune system and the tumour microenvironment (TME) across various cancer types [37]. Moreover, targeting mitochondrial complexes or related pathways represents a promising therapeutic strategy. For instance, Kwon et al. [38] developed a series of small molecules that successfully inhibited the activity of complex III (ubiquinol-cytochrome c reductase binding protein, UQBP), which resulted in reducing tumour growth in preclinical xenograft models (harbouring U87MG glioblastoma cells) with no detectable *in vitro* or *in vivo* toxicity observed. Further supporting the role of mitochondrial genes in cancer progression, several studies reported a significant increase in the expression of multiple mitochondrial genes—including MT-COI, MT-CYB, MT-ND1, and MT-RNR1—in colorectal cancer tissues compared with adjacent normal tissue [39].

In addition to the three mitochondrial genes, TUBA1A and H19 were also significantly downregulated following IL-17C stimulation. The TUBA1A gene encodes α-tubulin, which forms a dimer with β-tubulin to generate tubulin α1c (TUBA1C), a protein known to upregulate in various cancers and strongly correlate with poor survival [40]. The knockdown of TUBA1A appears to inhibit glioblastoma cell proliferation and invasion both *in vitro* and *in vivo* [40,41]. Additionally, the suppression of TUBA1B in colorectal cancer significantly reduced cancer cell viability and inhibited tumour cell proliferation [42].

Like TUBA1A, targeting H19 also appears to suppress glioma cell proliferation and enhance sensitivity to chemotherapy, thereby improving clinical outcomes [43]. Notably, Zhou et al. [44] demonstrated that H19 depletion led to a significant decrease in oral cancer cell proliferation and invasion. Similar findings were reported by Rajendran et al. [45], who demonstrated that H19 downregulation inhibited proliferation, invasion, migration, and the epithelial–mesenchymal transition in OSCC cells *in vitro*.

Gene set enrichment analysis (GSEA) revealed that IL-17C stimulation significantly enriched the oxidative phosphorylation (OXPHOS) and MYC gene sets. While OSCC cells primarily rely on glycolysis (the Warburg effect) rather than OXPHOS for energy production, forcing a shift from glycolysis to OXPHOS in highly glycolytic OSCC cells may disrupt their metabolic flexibility and inhibit tumour growth [46,47]. Interestingly, shifting metabolism away from glycolysis toward OXPHOS has been shown to suppress cancer cell proliferation, further supporting our findings [48].

Unexpectedly, IL-17C also enriched the MYC gene set, a well-known driver of cancer progression frequently overexpressed in HNSCC [49]. Whilst further studies are needed to understand why IL-17C induces MYC gene set activation, one possible explanation is that OSCC cells may attempt to restore glycolytic metabolism through MYC enrichment in response to the shift toward OXPHOS [50].

## Conclusions

**Fig. 7:**
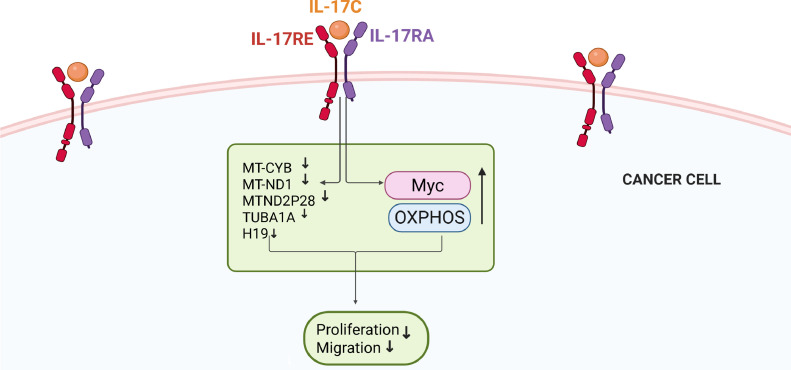
Schematic Model of IL-17C Signalling in OSCC Cells. IL-17C binds to the heterodimeric IL-17RA/IL-17RE receptor complex on the surface of OSCC cells, leading to the transcriptional downregulation of MT-CYB, MT-ND1, MTND2P28, TUBA1A, and H19, and the upregulation of the MYC and oxidative phosphorylation (OXPHOS) pathways. These molecular changes are associated with diminished cancer cell proliferation and migration, suggesting a tumour-suppressive role for IL-17C in OSCC.

In this study, we demonstrated that IL-17C regressed OSCC tumour growth both *in vitro* and *in vivo*. Our findings support the hypothesis that the IL-17C–IL-17RA/IL-17RE receptor complex may play a protective role in OSCC by downregulating MT-COI, MT-CYB, MT-ND1, MT-RNR1, TUBA1A, and H19, and by upregulating OXPHOS and the MYC gene sets (Fig. 7). These results suggest that IL-17C may inhibit cancer progression in a subset of OSCC patients and could serve as a promising candidate for targeted therapy for some OSCC cancer patients.

## Statements and declarations

### Funding

The authors gratefully acknowledge the funders of this study: the Research Council of Finland, the Sigrid Jusélius Foundation, the Minerva Foundation, and the Finnish Dental Society Apollonia. Open access funded by Helsinki University Library.

### Ethics approval

The cancer samples used to establish the OSCC cell lines were collected following institutional research ethics board approval (Regional Ethics Committee of Northern Ostrobothnia Hospital District, statement number 31/2016). Patient participation was voluntary and required informed consent.

## CRediT authorship contribution statement

**Zivile Giedraityte:** Writing – original draft, Methodology, Formal analysis, Data curation, Conceptualization. **Joosua Suominen:** Writing – original draft, Formal analysis, Data curation, Conceptualization. **Wafa Wahbi:** Writing – review & editing, Methodology, Formal analysis, Data curation. **Ahana Kapuge Dona Varuni Yashodha Ratnayake:** Writing – review & editing, Methodology, Data curation. **Tuulia Onali:** Writing – review & editing, Methodology, Data curation. **Martina Giacomini:** Writing – review & editing, Formal analysis, Data curation. **Sami Kilpinen:** Writing – review & editing, Formal analysis. **Katja Korelin:** Writing – review & editing, Data curation. **Maija Risteli:** Writing – review & editing, Data curation. **Tuula Salo:** Writing – review & editing, Investigation, Funding acquisition, Formal analysis, Data curation, Conceptualization. **Ahmed Al-Samadi:** Writing – review & editing, Supervision, Investigation, Funding acquisition, Formal analysis, Data curation, Conceptualization.

## Declaration of competing interest

The authors declare the following financial interests/personal relationships which may be considered as potential competing interests: Ahmed Al-Samadi reports financial support was provided by Research Council of Finland. Ahmed Al-Samadi reports financial support was provided by Sigrid Jusélius Foundation. Ahmed Al-Samadi reports financial support was provided by Minerva Foundation. Ahmed Al-Samadi reports financial support was provided by Finnish Dental Society Apollonia. If there are other authors, they declare that they have no known competing financial interests or personal relationships that could have appeared to influence the work reported in this paper.

## Data Availability

The datasets generated during and/or analysed as a part of the current study are available from the corresponding author upon reasonable request.

## Appendix Supplementary materials

Image, application 1
