# Supplementary material for: The protective role of IL-17C in oral squamous cell carcinoma
Source: Transl Oncol. 2025 Sep 19;62:102541. doi: 10.1016/j.tranon.2025.102541 (PMC12481104; doi:10.1016/j.tranon.2025.102541)
Supplement: Supplementary file 1 [file mmc1.docx]

**SUPPLEMENTS**

**Supplemental Fig. 1** Representative Images of UH-SCC-17B Spheroids (Days 1 to 4).


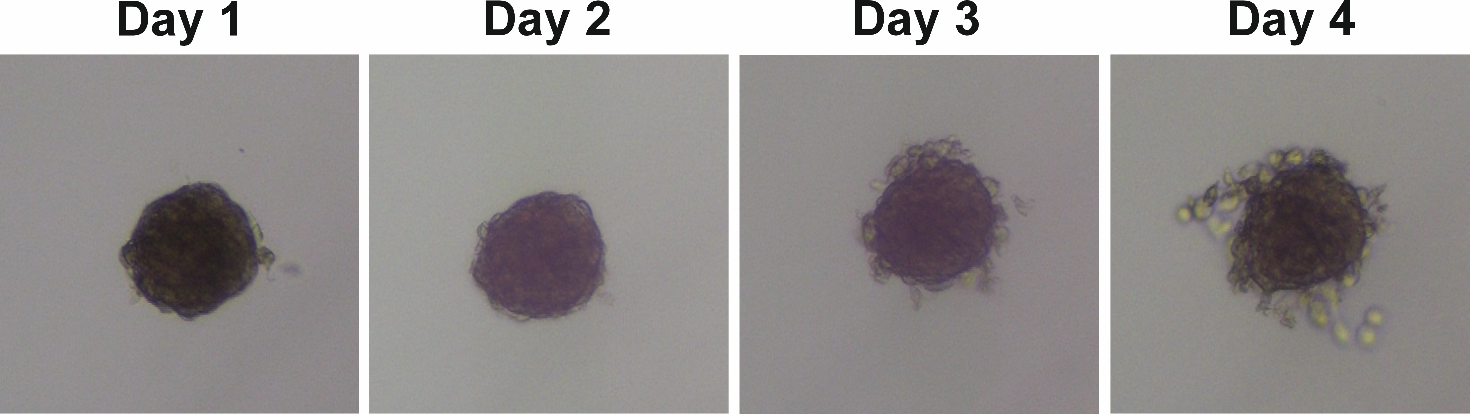


**Supplemental Table 1** Sequences of the Human Gene Primers Used.

| **Gene** | **Forward** | **Reverse** |
| --- | --- | --- |
| IL-17RA | 5′-CTGGTTCATCACGGGCATCTCC-3′ | 5′-GGTGGTCGGCTGAGTAGATGATC-3′ |
| IL-17RE | 5′-TCCTGGAATGTAAGCATGGATACC-3′ | 5′-GGAAGGGAATGATGAGGTCTAGTG-3′ |
| GAPDH | 5′-AAGGTCATCCCTGAGCTG-3′ | 5′-TGCTGTAGCCAAATTCGTTG-3′ |
